# How is the situation of women in leadership positions in medical education in Germany?

**DOI:** 10.3205/zma001557

**Published:** 2022-07-15

**Authors:** Inga Hege, Katrin Schüttpelz-Brauns, Claudia Kiessling

**Affiliations:** 1 University of Augsburg, Faculty of Medicine, Medical Education Sciences, Augsburg, Germany; 2 Heidelberg University, Medical Faculty Mannheim, Division for Study and Teaching Development, Medical Education Research Department, Mannheim, Germany; 3 Witten/Herdecke University, Faculty of Health, Education of Personal and Interpersonal Competencies in Health Care, Witten, Germany

**Keywords:** gender equality, mentoring, medical education

## Abstract

In Germany, about two thirds of students and doctoral candidates in medicine are female. The proportion is only about 35% for post-doctoral degrees and much lower for many leadership positions at medical schools and on medical education committees. Although reasons for this have long been known, changes are slow in coming. Therefore, with this commentary, we would like to shed light on the current situation regarding gender equality in Germany in medical education and identify and discuss measures. These include, for example, mentoring and networking programs as well as greater consideration of women in committees.

## Abstract (de)

In Deutschland sind etwa zwei Drittel der Studierenden und der Promovierenden in der Medizin weiblich. Bei den Habilitationen liegt der Anteil nur noch bei ca. 35% und bei vielen Leitungspositionen an Fakultäten und in Gremien der medizinischen Ausbildung deutlich darunter. Obwohl Gründe hierfür lange bekannt sind, kommen Änderungen nur langsam voran. Wir möchten daher mit diesem Kommentar die aktuelle Situation bezüglich der Gleichstellung in Deutschland in der medizinischen Ausbildung beleuchten und Maßnahmen aufzeigen und diskutieren. Dazu gehören z.B. Mentoring- und Vernetzungsprogramme sowie die stärkere Berücksichtigung von Frauen in Gremien.

## Introduction

“After all, there are no suitable women” is still a common response when it comes to filling committees and leadership positions in medicine or medical education.

Like in many other countries, more female than male students study medicine in German-speaking countries. For example, in the winter term 2020/2021, around two thirds of the approximately 100,000 medical students in Germany were female [1]. A similar distribution can be seen in the number of doctorates in medicine [2]. However, in 2020, the proportion of female habilitation candidates was only 35% [3], and the proportion of women holding professorships in Germany continues to be below 30% across all disciplines [4], [5]. The “gender pay gap” has also improved slightly in the recent years to an average of 18% but is still above the EU average of 15%. In the health and social care sector, the gender pay gap is with 24% [6] even larger and is also present in the salaries of professors in Germany [7].

The reasons for this are manifold and have been known for a long time. For example, stereotypes and prejudices often still exist when filling management positions with women, such as their assumed higher risk aversion or leaving the workplace for family reasons [8]. At the same time, women are less likely to apply for leadership positions – frequently cited reasons include a lack of protected time, support, experience, and mentors [9]. Also, men still have better networks than women and support each other in publishing articles or applying for research funds and thus improve their chances to be considered for an academic leadership position [10].

To meet these challenges, some measures have already been implemented at universities, such as mentoring programs tailored to the career planning of women or equality plans to ensure equal opportunities when filling leadership positions and professorships. There was also funding from the BMBF in 2020 for projects focusing on “women in science, research and innovation: making achievements and potential visible and structural anchoring of visibility” [11]. 

However, despite these measures, the proportion of women in leadership and committee positions in education and research, and thus their visibility, is still too low [12], [13]. 

With this commentary, we would like to draw attention to the current situation of women in committee and in leadership positions in medical education in German-speaking countries and identify possible starting points for more diversity. 

## Current situation in medical education in Germany

Key organizations in the orientation of research and teaching in the field of medical education in Germany are the Medical Faculty Association (MFT), the Society for Medical Education (GMA), the Association of the Scientific Medical Societies (AWMF), leadership positions (deans, deans of studies) of the faculties as well as the professorships and chairs for medical education. 

Based on our internet research (as of October 2021), the proportion of women in leadership positions at all these organizations is a maximum of 42%. The lowest proportion of women is in the AWMF presidium with 9% (n=1) and in the MFT presidium and board with 10% (n=1). The proportion of female deans or deans of studies at the 49 state and state-recognized medical faculties in German-speaking countries is slightly higher with 15.5% and 23.5%, respectively. Moreover, of the 11 chairs or professorships for medical education in German-speaking countries known to us, 36% (n=4) are occupied by women. 

On the GMA steering committee, the proportion of women rose from 25% to 42% after the last election in fall 2021. In GMA's 25 committees dedicated to the various topics of medical education, the proportion of women chairs is also around 40%, and among the vice-chairs, women are with 57% even in the majority.

At the GMS Journal for Medical Education (JME), the most important scientific German-language journal for medical education, the proportion of women on the editorial board is less than 20% and the two leadership positions are filled by men only [https://www.egms.de/en/journals/zma/about.htm#editorial]. In comparable international journals, such as BMC Medical Education [https://bmcmededuc.biomedcentral.com/about/editorial-board], the proportion of women is significantly higher.

## Outlook

The data presented show that the proportion of women in relevant leadership positions and committees in German-speaking countries in medical education remains low. 

People tend to promote people who are like them in the sense of the “younger self” [4], [14]. Therefore, we would like to promote a greater awareness for a balanced staffing of board, committee, and leadership positions. On both, the individual and structural levels, it must be possible in the future to establish gender-equitable participation by creating appropriate framework conditions and resources. The statement “there are no suitable women” should lead to an analysis and adjustment of structural framework conditions, e.g., tendering procedures, composition of appointment committees or meeting times. 

On the other hand, we would like to encourage women in medical education to increase their networking activities and apply confidently for leadership positions and committees. Possibly, the introduction of a quota could also be a supporting measure for committees with a very low proportion of women, such as the board and the presidium of the MFT. 

At its General Assembly in 2019, the German University Rectors’ Conference (HRK) proposed using the experience and networks of “elder stateswomen” [13]. This can be achieved with a mentoring and networking program to make role models visible and to support young women in a targeted manner and as early as possible to qualify and apply for leadership positions in the field of medical education. In addition to this measure, Tricco et al. as well as the German Council of Science and Humanities suggest further measures such as a quota-based staffing of leadership positions, gender-sensitive language, the formulation of goals to be achieved regarding existing disparities, a stronger involvement of women in review and recruitment procedures, or a gender bias training [14], [15]. 

We are aware that a balanced mix of men and women on boards and in leadership positions is only one aspect of more diversity. The same applies to people who do not wish to be assigned to one of the binary genders, people with disabilities, or people with a migration background. We advocate more diversity here in every respect as greater diversity in teams leads to fewer mistakes, better thought-out and more objective decisions, and innovative ideas [16], [17]. 

We see it as an important mission that concerns us all and therefore needs to be supported in organizations such as the GMA and the faculties. With this commentary, we would like to emphasize our recommendation for the establishment of a mentoring and networking program within the GMA and hope that in the future we will discuss the aspects raised at all levels.

## Author contributions

All three authors contributed equally.

## Competing interests

The authors declare that they have no competing interests. 

## References

[1] Statistisches Bundesamt (2021). Anzahl der Studierenden im Fach Humanmedizin in Deutschland nach Geschlecht in den Wintersemestern von 2007/2008 bis 2020/2021.

[2] Statistisches Bundesamt (2021). Frauen promovieren häufiger in Gesundheitswissenschaft und Humanmedizin.

[3] Statistisches Bundesamt (2021). Mehr Habilitationen von Frauen im Jahr 2020.

[4] Hibbeler B, Korzilius H (2008). Arztberuf: Die Medizin wird weiblich. Dtsch Arztebl.

[5] Statistisches Bundesamt (2022). Frauenanteil in der Professorenschaft in Deutschland im Jahr 2020 nach Bundesländern.

[6] Statistisches Bundesamt (2021). Gender Pay Gap.

[7] Deutscher Hochschulverband (2020). Geschlechterunterschiedliche Vergütung in der Wissenschaft.

[8] Littmann-Wernli S, Schubert R (2001). Frauen in Führungspositionen - Ist die “gläserne Decke” diskriminierend?. Z Arbeit.

[9] Esparza R, Stanford FC, Silver JK (2021). How Can More Women be Elected to Leadership Positions in Medical Specialty Societies?. Acad Med.

[10] Witte F (2018). Dünne Luft- Ärztinnen in Führungspositionen sind in Deutschland selten. Alte Rollenbilder, Familie wie ein vom Spardruck gezeichnetes Gesundheitswesen bremsen den Aufstieg. Süddtsch Z.

[11] Bundesministerium für Bildung und Forschung (2020). Richtlinie zur Förderung von Projekten zum Themenschwerpunkt „Frauen in Wissenschaft, Forschung und Innovation: Leistungen und Potenziale sichtbar machen, Sichtbarkeit strukturell verankern“ („Innovative Frauen im Fokus“).

[12] Alwazzan L, Al-Angari SS (2020). Women’s leadership in academic medicine: a systematic review of extent, condition and interventions. BMJ Open.

[13] Hochschulrektorenkonferenz (2019). Frauen in Leitungspositionen in der Wissenschaft - Entschließung der 27.

[14] Wissenschaftsrat (2007). Empfehlungen zur Chancengleichheit von Wissenschaftlerinnen und Wissenschaftlern.

[15] Tricco AC, Bourgeault I, Moore A, Grunfeld E, Peer N, Straus SE (2021). Advancing gender equity in medicine. CMAJ.

[16] Rock D, Grant H (2016). Why Diverse Teams Are Smarter. Harvard Business Rev.

[17] Díaz-García C, González-Moreno A, Sáez-Martínez FJ (2013). Gender diversity within R&D teams: Its impact on radicalness of innovation. Innovation.

